# Synthetic cells for phage therapy: a perspective

**DOI:** 10.3389/fcimb.2025.1690404

**Published:** 2025-10-22

**Authors:** Vishwesh Kulkarni, Nadanai Laohakunakorn, Sahan B. W. Liyanagedera

**Affiliations:** ^1^ School of Biomedical Engineering and Imaging Sciences, King’s College London, London, United Kingdom; ^2^ SUSTech-King’s School of Medicine, Shenzhen, China; ^3^ Institute of Quantitative Biology, Biochemistry and Biotechnology, University of Edinburgh, Edinburgh, United Kingdom; ^4^ Biophoundry Inc., Chapel Hill, NC, United States

**Keywords:** cell-free protein synthesis, transcription/translation (TXTL), synthetic cells, bacteriophages, phage therapy, hydrogel

## Abstract

A synthetic cell is a membrane-bound vesicle that encapsulates cell-free *transcription/translation* (TXTL) systems. It represents a transformative platform for advancing bacteriophage therapy. Building on experimental work that demonstrates (i) modular genome assembly, (ii) high-yield phage TXTL systems, and (iii) smart hydrogel encapsulation, we explore how synthetic cells can address major limitations in phage therapy. The promising advances include point-of-care phage manufacturing, logic-responsive antimicrobial biomaterials, and new chassis to dissect the dynamics of phage-host interactions. We also propose a roadmap for the deployment of synthetic cells as programmable and evolvable tools in the context of laboratory research and translational clinical adoption.

## 1 Introduction

Phage therapy refers to the therapeutic use of bacteriophages—viruses that specifically infect and kill bacteria—to treat bacterial infections (Skurnik et al., 2025). Although it has been practiced for over a century in limited clinical settings, its widespread authorized adoption remains restricted, with most applications occurring under compassionate use or experimental frameworks (Yang et al., 2023). This is largely due to regulatory uncertainty, challenges in large-scale manufacturing and robust engineering methods for personalized phage cocktail design (Zalewska-Piątek 2023).

**Figure 1:**
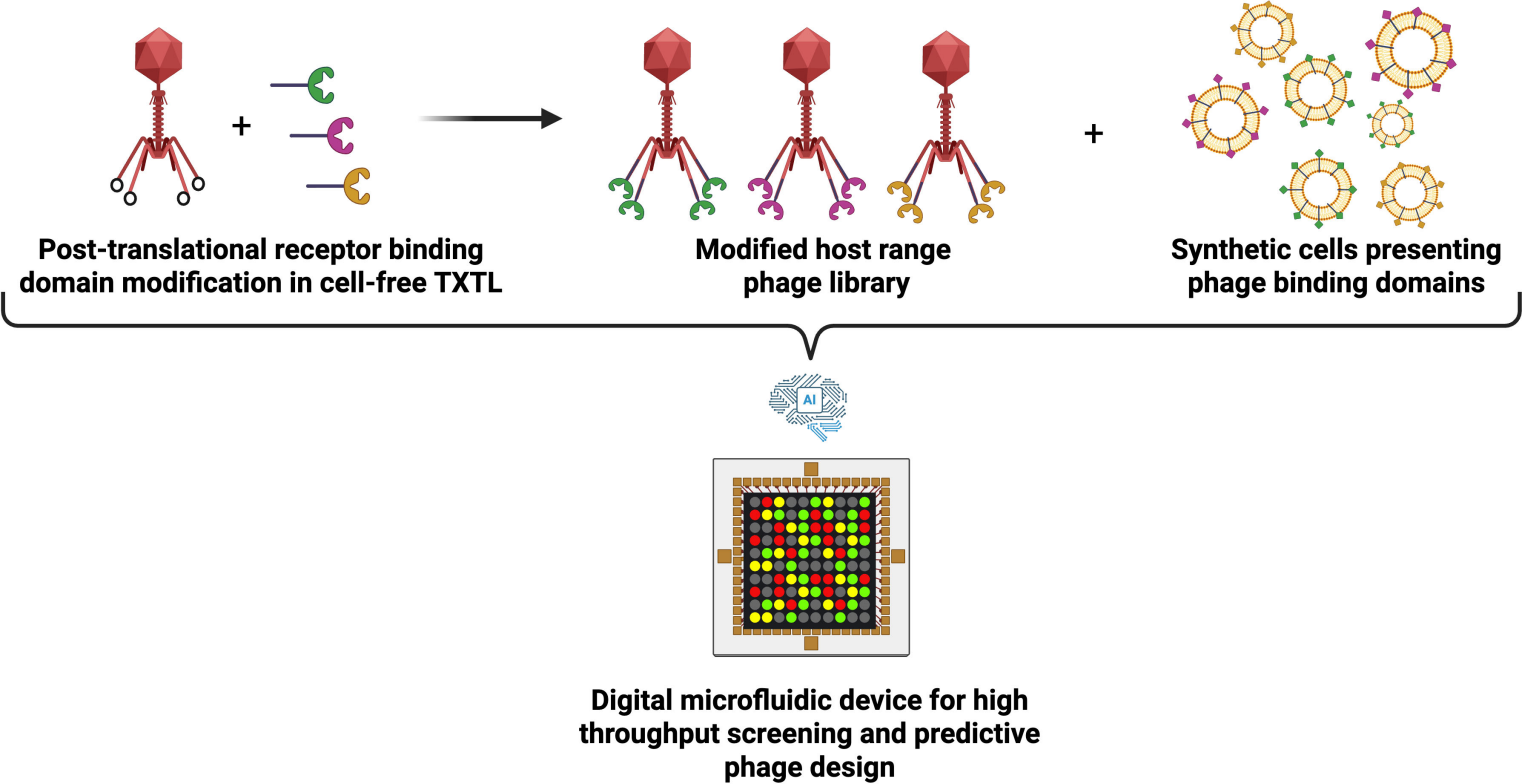
High-throughput synthetic cell and engineered phage interaction platform for predictive phage design. Schematic overview of a proposed library on library screening framework. Scaffold phage are assembled in TXTL and post-translationally decorated with receptor binding domain modifications to create a modified host range phage library. Synthetic cells, each engineered to express and present distinct receptor landscapes are combined with diverse host range phage library using combinatorial digital microfluidics. These high throughput experiments generate large interaction data sets that capture receptor-phage compatibility and infection outcomes. Data sets can be used to train advanced machine learning models for predictive phage design.

However the recent resurgence of phage therapy, catalyzed by advances in synthetic biology, has equipped us with an expanding arsenal of engineered bacteriophages to combat the *antimicrobial resistance* (AMR) crisis (Sawa et al., 2024). It is now possible to reprogram the phage-host range (Ando et al., 2015), enhance lytic activities (Son et al., 2021), and even assemble entire phage particles at clinically relevant titres *in vitro* through the use of cell-free transcription-translation (TXTL) systems (Garenne et al., 2021; Liyanagedera et al., 2022b; Levrier et al., 2024). However, the next great leap in phage therapeutics may not lie solely in the phage itself, but in the platforms used to produce, deploy, and study them. *Synthetic cells*, defined here as vesicles that encapsulate the TXTL machinery, offer exciting opportunities (Garamella et al., 2019a). Our exposition in this paper goes beyond the *in vitro* use of cell-free TXTL systems and is inspired by early work in the fields of artificial life and synthetic cells [see (Adamala et al., 2017; Bayoumi et al., 2017; Lentini et al., 2017; Majumder et al., 2017; Rampioni et al., 2018; Sakamoto et al., 2018; Tang et al., 2018)]. We propose that programmable modular synthetic cells capable of assembly of infectious phage particles or phage-associated proteins (i.e., lysins/de-polymerases) could serve as a powerful enabling technology for advanced phage therapy. Positioned as a conduit for *artificial intelligence* (AI) driven phage discovery, synthetic cells can provide the essential experimental infrastructure to generate large, diverse, and discrete datasets suitable for training the underlying machine learning models (**Figure 1**). For example, such an experimental platform capable of high-throughput screening could generate data sets to predict phage-host interactions, enabling forward engineering of phage therapies against bacterial pathogens. Thus, synthetic cells can serve as a platform that will not only enable new therapeutic strategies, but also unlock new ways to probe the fundamental biology of the interactions between phage and host (Rothschild et al., 2024).

## 2 Cell-free TXTL and synthetic cells

Cell-free TXTL is an *in vitro* platform that reconstitutes the core molecular machinery of cellular transcription and translation, supported by energy regeneration and co-factor recycling, without the constraints of a cell membrane (Noireaux et al., 2003). This technology uses a cytoplasmic extract, typically derived from *Escherichia coli* (*E. coli*), which provide the core transcription-translation machinery, including RNA polymerase, ribosomes and translation factors, along with endogenous enzymes that support energy regeneration and central metabolism. By supplementing this extract with exogenous DNA template(s) and energy sources, TXTL enables robust synthesis of proteins and complex genetic circuits in a one-pot reaction, offering unparalleled speed, flexibility, and control for synthetic biology, functional genomics, and bio-manufacturing.

Synthetic cell construction integrates cell-free protein synthesis and liposome technology to create artificial bioreactors that replicate cellular phenomena. An increasingly large number of studies have recently demonstrated how synthetic cell technology has reached a level of complexity that could make them useful as actuators and mediators of signal transduction (Adamala et al., 2017; Bayoumi et al., 2017; Lentini et al., 2017; Majumder et al., 2017; Rampioni et al., 2018; Sakamoto et al., 2018; Tang et al., 2018). Quite a few recent studies demonstrate cell-free expression-mediated communication between synthetic cells and bacteria (Lentini et al., 2017; Rampioni et al., 2018), as well as between synthetic cells themselves (Adamala et al., 2017; Bayoumi et al., 2017; Tang et al., 2018). Most of such studies have established robust and replicable unidirectional communication. However, a study has also described bidirectional communication; where synthetic cells ‘translated’ chemical signals that cannot be sensed naturally, into perceivable inputs for *E. coli* (Lentini et al., 2014). Furthermore, membrane channels, such as alpha-hemolysin pores, can be used to decorate the phospholipid bilayer of the liposome. Such channels can either be incorporated during the construction of the liposome or be expressed *in situ* in response to a defined stimulus (Majumder et al., 2017). As such, through the coupling of synthetic genetic regulatory mechanisms to protein production, synthetic cells can be made to perform as highly programmable units of artificial life (Adamala et al., 2017).

## 3 Synthetic cells as phage research models

By recreating selected features of natural infection cycles in a controlled environment, synthetic cells provide a tractable platform to dissect phage-host interactions, test mechanistic hypotheses, and build a modular toolkit for phage research. This research-oriented perspective positions synthetic cells as reductionist experimental models to explore fundamental questions in virology and synthetic biology. A key advance in the use of synthetic cells for phage therapy is the recent demonstration of a fully synthetic cell-free phage infection cycle as reported in (Levrier et al., 2025). Here, the researchers created liposome-based synthetic cells encapsulating an *E. coli* cell-free TXTL system, with a *lipopolysaccharide* (LPS) outer shell. The LPS outer shell enabled T7 phage attachment, infection and delivery of the T7 phage genome into the synthetic cell. Following infection, the encapsulated TXTL machinery was able to act on the delivered phage genome and produce phage particles within synthetic cells. This “all-cell-free viral cycle” effectively mimics phage infection by facilitating attachment, viral genome replication, and phage production within discrete synthetic compartments. In this platform, the progeny of the phages was released via osmotic shock, an external trigger that disrupted the synthetic cell membrane, liberating the newly assembled virions. To advance toward truly autonomous systems, programmable lysis mechanisms are required, and here the bacteriophage holin proteins could be the key.

Holin proteins are an integral part of the lytic cycle and function as a “molecular clock” to time the lysis of the host (Schwarzkopf et al., 2024). These proteins, which when expressed from the phage genome, progressively accumulate in the membrane of the bacteria and undergo a sudden oligomerization to form large holes in the cytoplasmic membrane, thus providing access to phage-encoded endolysins to attack the cell wall. These coordinated events result in instantaneous lysis of the bacterial host, thus releasing the now assembled phage particles to enable another round of the lytic cycle.

A liposome-based single bilayer synthetic cell can be considered as a model of the cytoplasm and the cytoplasmic membrane of the bacterium (or a bacterium without a cell wall). In fact, early work on putative holin characterization used classical liposome fluorescence leakage assays to determine the lytic potential of the protein in question (Smith et al., 1998). Taking inspiration from the natural mechanisms of holing function, proof of concept studies have attempted programmable triggered release of phage assembled within synthetic cells. For example, initial attempts encapsulated TXTL systems capable of K1F phage assembly in synthetic cells based on single bilayer liposomes produced by the emulsion transfer technique (Liyanagedera 2022a). In this approach, the purified K1F phage genomes were co-encapsulated with the cell-free TXTL machinery during vesicle formation, rather than being introduced by infection. These synthetic cells exhibited dramatic loss of vesicles after 12 hours of incubation, likely due to premature holin-mediated lysis before phage assembly was complete. In contrast, controls with transcription inhibited by rifampicin remained morphologically stable. Consistent with this, plaque assays revealed that while bulk TXTL reactions produced phages, encapsulated systems did not, highlighting the challenge of synchronizing phage assembly with timed release. In addition, protein expression yields achieved per particle in encapsulated cell-free reactions are typically lower than those of bulk systems, likely reflecting the limited energy reserves within individual compartments (Noireaux and Libchaber 2004; Gonzales et al., 2022). This constraint becomes particularly relevant for larger phages, such as those typically used in phage therapy, whose assembly places greater energetic demands on the encapsulated system (Mahmoudabadi et al., 2017).

Despite these advances, significant challenges remain before synthetic cells can serve as robust models of phage infection. Current systems are constrained by their reliance on *E. coli*-derived TXTL, restricting applicability to *E. coli*-infecting phages (Garenne et al., 2021). To broaden the scope, it is necessary to develop non-model cell-free systems derived from other bacteria capable of phage assembly, which would allow the study and production of phages that infect diverse hosts (Brooks et al., 2023; Emslander et al., 2022). Similarly, while LPS coated vesicles enable T7 infection, many phages depend on alternative receptors such as outer membrane proteins or capsular polysaccharides, as exemplified by K1F phage (Liyanagedera et al., 2022b. Addressing this limitation may involve membrane engineering approaches, such as functionalizing synthetic cell membranes with purified receptors (Elani 2016) or integrating *outer membrane vesicles* (OMVs) from non-model bacteria to recreate authentic attachment environments (Bali et al., 2023).

## 4 Smart antimicrobial materials

Synthetic cells are emerging as minimalist and programmable bioreactors for the *de novo* synthesis and delivery of biological drugs (Monck et al., 2024). This approach goes beyond traditional drug delivery by creating systems capable of sensing, decision making, and autonomous action at the infection site (Gispert and Elani 2025).

Although synthetic cells show promise as standalone antimicrobial agents, their therapeutic potential is magnified when integrated into structured materials. The smart antimicrobial material, i.e. a material that can (i) sense a bacterial threat, (ii) synthesize phage on demand, and (iii) release those phages at the site of infection, is a compelling goal for phage therapy (Beth et al., 2023; El-Husseiny et al., 2022). Its foundation lies at the convergence of smart materials and artificial life.

The bottom-up design of synthetic cells and their translation into the creation of smart antimicrobial materials is reliant on compartmentalized electrical or chemical signal transduction in which the complex tissue is realized by interconnecting the “blocks” or “compartments” of well-characterized synthetic biological circuits (Rothschild et al., 2024). From the perspective of a smart material, the compartmentalization of synthetic biology modules allows spatial and temporal control over the properties of the material, thus enabling the same material to have defined regions with different capabilities (Kusters et al., 2011; Lentini et al., 2016; Stadler et al., 2009).

Critical innovation would arise from the incorporation of genetic circuits designed to respond to pathogenic stimuli. These circuits are engineered to detect quorum-sensing molecules (e.g. acyl-homoserine lactones from Gram-negative bacteria), virulence factors, or specific microenvironmental pH changes associated with biofilm formation. An envisioned use case is as follows. Upon detection of these *pathogen associated molecular patterns* (PAMPs), the genetic circuit of the synthetic cell will be activated, thereby triggering the expression and synthesis of bacteriophage, *antimicrobial peptides* (AMPs), or bacteriolytic enzymes (e.g., lysins) directly within the synthetic compartment. AMPs, such as melittin or defensin analogs, are particularly suited for this application because of their broad-spectrum activity and the difficulty pathogens face in developing resistance against them. The synthesized AMPs can accumulate within the vesicle until membrane lysis releases a bolus, or they can be secreted through engineered pore proteins (e.g., alpha-hemolysin nanopores) for sustained controlled release. The “smart” functionality would be embedded in the feedback and regulatory mechanisms of the genetic circuitry. Several excellent results are available on the design of such circuits [see (Brophy and Voigt 2014; Nielsen et al., 2016; Palacios et al., 2025), and references therein].

A more advanced application involves programming synthetic cells for the synthesis and self-assembly of antimicrobial nanostructures. This could involve the expression of enzymes that catalyze the precipitation of toxic metal ions (e.g., silver) into nanoparticles directly on the bacterial membrane or the production of self-assembling peptide nanofibers that disrupt biofilm integrity. The primary challenges remain to optimize the stability, yield, and kinetic parameters of the encapsulated TXTL system, preventing immune recognition *in vivo*, and scaling production.

**Figure 2:**
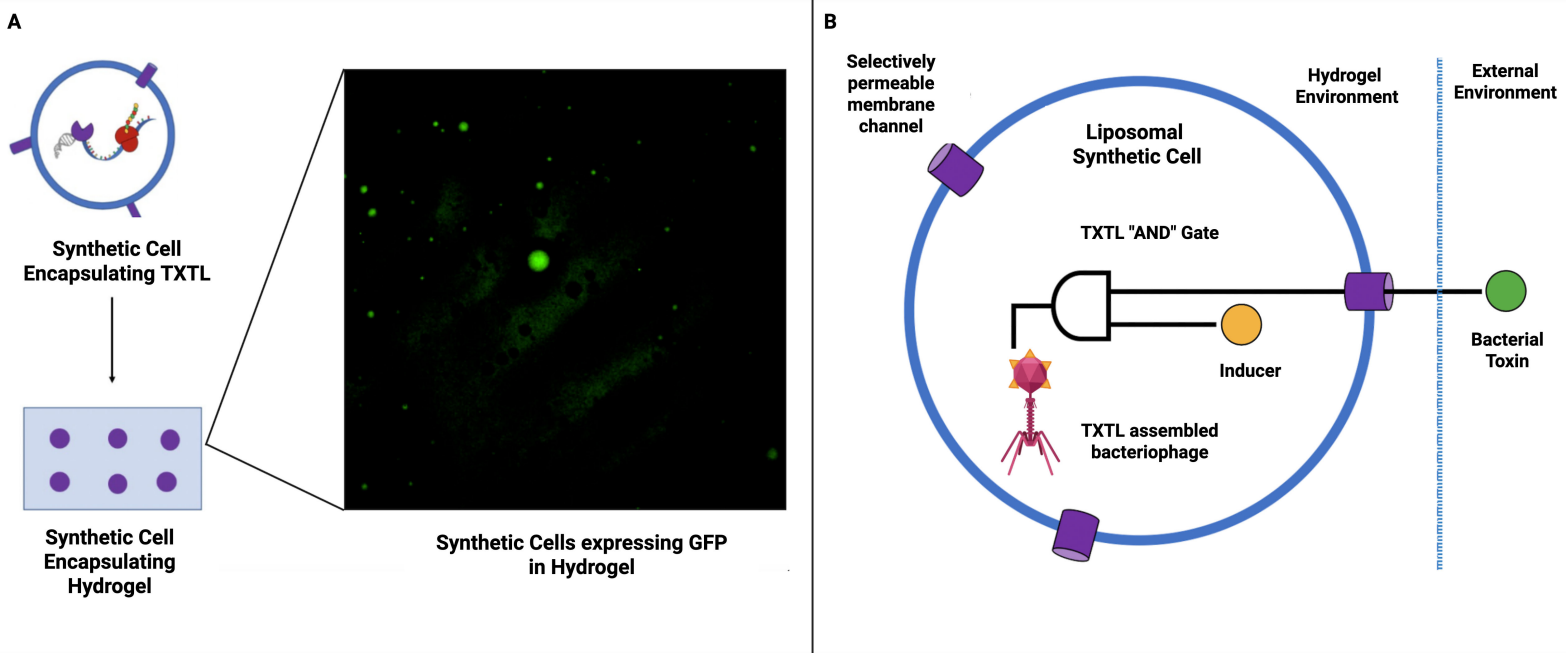
Synthetic cells for smart antimicrobial materials. **(A)** Synthetic cells expressing GFP immobilized within hydrogel (Liyanagedera (2022a). These synthetic cells are immobilized within a hydrogel matrix, thus paving the way for the utilization of synthetic cells to create smart hydrogel-based materials. **(B)** Schematic representation of smart antimicrobial material proof of concept. A hydrogel-based material is used to encapsulate synthetic cells containing a simple TXTL “AND” gate. The circuit will produce an output (cell-free assembled bacteriophage) when activated by an inducer molecule (trapped inside the synthetic cell) and a bacterial toxin which enters through a selectively permeable membrane.

Building on these seminal works, we can envision a proof-of-concept for a smart antimicrobial hydrogel material wherein a population of synthetic cells could be engineered to sense the presence of bacteria and, in response, produce and release a cell-free assembled phage (**Figure 2**). To mitigate the challenge of autolysis of synthetic cells producing phage outlined previously, one could exercise holin expression control using antisense ssDNA to silence translation, allowing tunable delays in lysis (Vogele et al., 2021). Trigger plasmids could offer further control by releasing silenced mRNA in response to stimuli, such as quorum-sensing molecules, enabling infection-responsive executioner cells (Garamella et al., 2019b). Such strategies would enable the ultimate aim of the construction of autonomous antimicrobial modules capable of both sensing bacterial presence and responding with a precise therapeutic payload (Liyanagedera 2022a). Furthermore, they could allow the implementation of prolonged smart functionality that does not require a gel-sol transition (semi-solid gel to liquid-like sol) and thus preserve intelligence and material integrity.

Nonetheless, a significant technical challenge in the construction of any smart material is the movement of molecules within it (Badeau et al., 2018). This directly impacts the embedding of TXTL circuitry within a material. Specifically, a hydrogel architecture would have to strike a balance between the permeation of signal inputs and the leaching out of the TXTL machinery, as well as any desired structural properties of the hydrogel. Methods for the encapsulation of logic circuitry in extracellular compartments are not available today but synthetic cells that compartmentalize cell-free reactions might resolve this difficulty (Adamala et al., 2017).

## 5 Future directions: a unified platform for phage therapy

In light of the continued progress of synthetic biology research, synthetic cells could soon be engineered to support the entire phage life cycle *in vitro*. By modularly integrating genome assembly cassettes, capsid display systems, and programmable lysis circuits, researchers can reconstruct phage behavior from the ground up. These platforms would enable systematic investigation of key parameters such as tail fiber specificity, lysis timing, and CRISPR evasion within a fully defined, controllable context. For phage therapy, these synthetic cells will offer a highly useful platform to investigate fundamental questions in phage biology (Rothschild et al., 2024; Sampson et al., 2024).

A promising direction is the development of a modular synthetic cell library that features a variety of bacterial surface receptors. This would enable high-throughput compatibility testing with engineered phage libraries, facilitating large-scale screening of phage–host interactions. Even today it is known that beyond the facilitation of simple binding assays, these synthetic cells also offer a unique opportunity to dissect the molecular mechanics of infection (van den Berg et al., 2022). For example we envision that the *internal capsid protein* (ICP) ejection process, which is central to the delivery of the phage genome and has been exploited for diagnostic applications, can be explored in isolation. By co-encapsulating purified “sentinel” phages with bacterial components such as membrane receptors or cell wall fragments, researchers can pinpoint precise triggers for DNA and ICP release, all without the complexity of live cell systems.

These platforms could be combined with minimal TXTL systems such as the PURE (Protein synthesis Using Recombinant Elements) system, and further optimized with metabolic intermediates identified through virocell profiling, to enable fine-tuned emulation of host-like conditions (Yadav et al., 2025; Emslander et al., 2022). This bottom-up approach can help us answer fundamental questions such as, ‘*What is the minimal set of host factors required to assemble a phage?*’ The solution to this question will accelerate the development of GMP-compliant, minimal TXTL systems optimized for therapeutic phage production (Liyanagedera et al., 2022b).

Local expression of therapeutic payloads within synthetic cells offers advantages beyond serving merely as release vehicles: it enables on-demand co-synthesis of phages with unstable or short-lived compounds, provides precise spatiotemporal control of drug release (Monck et al., 2024), and reduces the risk of premature drug degradation prior to delivery (Kim et al., 2025). Nonetheless, in the context of *in vivo* therapeutic applications, additional challenges arise around stability, immunogenicity, and delivery of the synthetic cells themselves (Kim et al., 2025). In particular, early work on intratumoral protein synthesis using synthetic cells highlighted immunogenic risks associated with extract-derived endotoxin release (Krinsky et al., 2018), but recent advances in developing endotoxin free cell-free systems and advanced liposomal stabilisation strategies, are helping to mitigate these concerns (Wilding et al., 2019; Beltrán-Gracia et al., 2019; Meyer et al., 2025).

The power of synthetic cells will emerge when the capabilities of therapeutic production and mechanistic investigation are combined. Such a platform will not only manufacture a therapeutic phage but also test its efficacy and study its infection dynamics, establishing a rapid, iterative design–build–test–learn cycle entirely *in vitro*. A novel phage genome could be designed *in silico*, the corresponding DNA parts synthesized, assembled, encapsulated and introduced into a synthetic cell library that mimics various host profiles, all within a single modular framework.

The true programmability will be determined by what else is included in the encapsulated TXTL reaction. By co-encapsulating a scaffold phage genome with plasmids encoding various different *receptor binding domains* (RBDs) or other therapeutic payloads, it would be possible to dictate the final form of the phage post-translationally, thereby turning each synthetic cell into a bespoke production line (Liyanagedera et al., (2022b). Then, a single scaffold genome can yield a multitude of phenotypically distinct phage variants, with the final output determined simply by the “instruction set” of DNA parts included in the initial reaction.

With the incorporation of artificial intelligence (AI) and microfluidics, this vision can be scaled further: library-on-library interactions between engineered phages and synthetic cells that feature variable receptor landscapes could be rapidly screened (York 2025). The resulting data would make it feasible to build reliable and powerful machine learning models for predictive phage design, enabling automated workflows for personalized and precision-engineered phage therapies, as noted in (Keith et al., 2024).

Seminal work recently demonstrated that generative genome language models can design completely new phages with improved fitness and the ability to overcome resistance (King et al., 2025). Synthetic cells could provide an important complement to such AI-driven therapeutic phage design, providing reductionist experimental platforms for generating training data for phage-host interactions that remain unrepresented in public genome collections. Such approaches could fill a gap by providing experimentally validated data sets in areas where models otherwise face sparse or biased training data. Looking forward, ensuring that such interaction data sets are made publicly available - for example through national initiatives such as the UK’s open data programs for biomedical AI - will be critical in accelerating progress towards predictive phage therapy.

## Data Availability

The original contributions presented in the study are included in the article/supplementary material. Further inquiries can be directed to the corresponding author.
